# Overcoming resistance in advanced urothelial carcinoma: mechanisms of escape from antibody-drug conjugates and FGFR3 inhibition

**DOI:** 10.3389/fonc.2025.1654771

**Published:** 2025-12-02

**Authors:** Brandon Wummer, Michael Schwartz, Jordan Ciuro, Shahid Ahmed, Shreyas S. Joshi, Vikram M. Narayan, Bradley C. Carthon, Mehmet Asim Bilen, Jacqueline T. Brown

**Affiliations:** 1 Department of Medicine, Emory University School of Medicine, Atlanta, GA, United States; 2 Emory University Winship Cancer Institute, Atlanta, GA, United States

**Keywords:** advanced urothelial carcinoma, antibody-drug conjugates, FGFR3 inhibition, drug resistance, immunotherapy combined therapy

## Abstract

For decades, platinum chemotherapy was the mainstay of treating metastatic urothelial carcinoma (mUC). More recently, checkpoint inhibitors (CPI) were an important addition to the armamentarium capable of inducing durable responses for a minority of patients. Management of mUC has changed significantly with the advent of antibody-drug conjugate (ADC) therapies and fibroblast growth factor receptor inhibitors (FGFRi). Enfortumab vedotin, a Nectin-4 targeting ADC, is now the first line therapy of choice in combination with pembrolizumab. Erdafitinib, a pan FGFR1–4 inhibitor, is approved for patients with susceptible FGFR3 alterations. There are multiple other agents in development within both therapeutic classes that hold promise. But most patients will still succumb to their disease, either via primary or secondary resistance. This review looks critically at the approved and pipeline ADC and FGFR-targeting agents of interest in mUC as well as known mechanisms of resistance by which their efficacy is dampened. We propose strategies for overcoming resistance including combination strategies, tumor microenvironment modification, and drug structure modification to maximize efficacy. The progress to date in mUC has been remarkable, but there is still significant work to do in this deadly disease and this review highlights the gap between current available therapeutics and cure that so desperately needs to be closed.

## Introduction

Urothelial carcinoma (UC) is the ninth most common cancer in the world with 550,000 new cases annually (1). Historically, platinum-based chemotherapy has been the standard treatment for metastatic UC (mUC). However, more than half of patients with mUC were ineligible for cisplatin therapy and those who received treatment ultimately developed progression of their disease (2, 3). The treatment landscape evolved with the introduction of checkpoint inhibitors (CPI), initially used as monotherapy for cisplatin-ineligible or relapsed patients. Despite these advances, response rates to CPI monotherapy remain modest, ranging from 15 to 21%, with only a small subset of patients achieving a durable benefit (4). In response, CPI was used as maintenance therapy following platinum chemotherapy, and more recently in combination with chemotherapy followed by continued maintenance (5–8).

**Figure 1:**
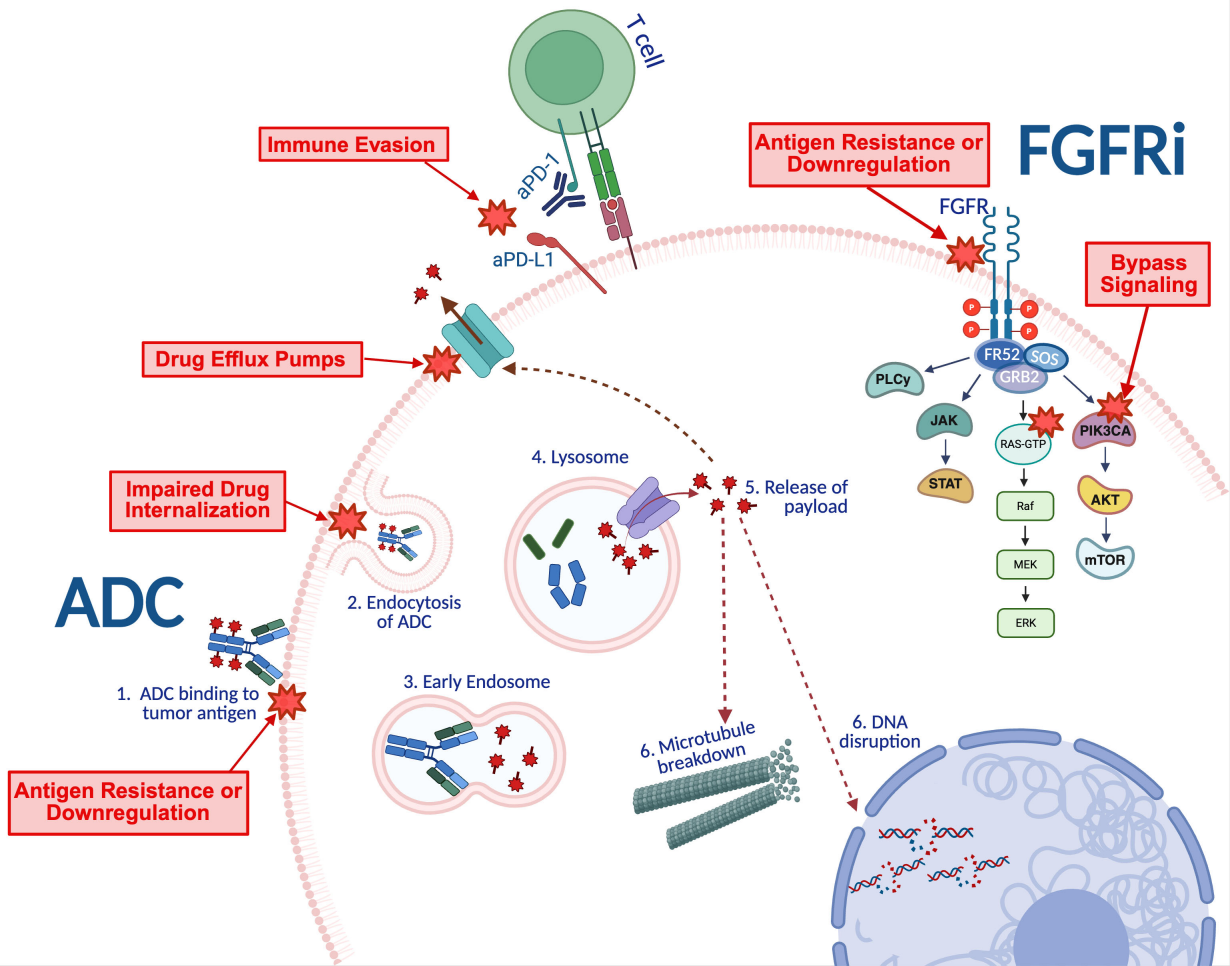
Mechanisms of action for antibody-drug conjugate (left) and FGFR inhibitor (right) therapies and corresponding mechanisms of resistance.

The emergence of antibody-drug conjugates (ADCs) and targeted therapies has reshaped the therapeutic paradigm in treating mUC. ADCs consist of three components: an antibody that binds target antigens expressed on tumor cells, a small molecule cytotoxic drug payload, and a linker molecule. After binding to its target tumor antigen, ADCs are endocytosed into tumor cells, processed and unlinked within lysosomes, releasing the payload and leading to cell cycle arrest through direct cytotoxicity. In contrast, targeted therapies act directly on intracellular signaling pathways that drive tumor progression (**Figure 1**).

Enfortumab vedotin (EV) is an ADC that targets nectin-4, a cell surface adhesion protein expressed on most urothelial carcinoma cells (9). When combined with pembrolizumab, a PD-1 inhibitor, EV has significantly improved clinical outcomes in patients with treatment-naïve mUC, nearly doubling the progression-free survival (PFS) and overall survival (OS) when compared to platinum-based chemotherapy (10). This combination has since been approved as first-line therapy in mUC regardless of cisplatin eligibility and is the new standard of care for most patients with mUC. Other ADCs currently approved for use in mUC include trastuzumab deruxtecan (T-DXd), a Her2-targeting ADC with a tissue-agnostic approval for HER2-expressing tumors, including UC (11). Sacituzumab govitecan, an anti-Trop-2 ADC that previously held an accelerated FDA approval in mUC based on a promising phase 2, single arm trial later lost this designation after the confirmatory phase 3 study showed no improvement in OS over single-agent chemotherapy. Disitamab vedotin is a HER2-targeting ADC of great interest in the pipeline, as are the Trop-2 targeting datopotamab deruxtecan and sacituzumab tirumotecan which have showed promise in early-phase clinical trials (**Table 1**).

**Table 1:** Selected trials and outcomes for antibody-drug conjugates (ADCs) and FGFR inhibitors in advanced UC.

Drug	Trial	NCT	Phase	Population	N (enrolled pts)	Control	ORR, % (95% CI)	mPFS, mo (95% CI)	mOS, mo (95% CI)
Antibody-drug conjugates									
Enfortumab vedotin (EV)	EV-101	NCT02091999	I	≥ 1 prior chemo and/or CPI	155	NA	43	5.4 (5.1- 6.3)	12.3 (9.3- 15.3)
	EV-201	NCT03219333	II	Cisplatin-ineligible, prior CPI	91 (cohort 2)	NA	52 (41.0- 62.0)	6.7 (5.0 - 8.3)	16.1 (11.3- 24.1)
	EV-301	NCT03474107	III	Prior platinum and CPI	608	Chemotherapy^*^	40.6 (34.9- 46.5)	5.6 (5.3-5.8)	12.9 (1.6-15.2)
EV + pembrolizumab	EV-103	NCT03288545	Ib/II	Cisplatin-ineligible, 1L	45	NA	73.3 (58.1- 85.4)	12.3 (8.2- NE)	26.1 (12.5- NE)
	EV-302	NCT04223856	III	1L	886	Platinum doublet	67.7 (63.1- 72.1)	12.5 (10.4- 16.6)	31.5 (25.4- NE)
Sacituzumab govitecan	TROPHY-U-01	NCT03547973	II	Prior platinum and CPI	113 (cohort 1)	NA	28.0 (20.2-37.6)	5.4 (3.5- 6.9)	10.9 (8.9-13.8)
	TROPiCS-04	NCT04527991	III	Prior platinum and CPI	711	Chemotherapy^*^	23.0 (18.0-27.0)	4.2 (3.7- 4.9)	10.3 (8.8-11.6)
Sacituzumab tirumotecan	MK-2870-001	NCT04152499	I/II	2L	11	NA	45.5 (16.7-76.6)	5.8 (1.7- NE)	NE (2.0- NE)
				3L+	38		26.3 (13.4-43.1)	5.0 (3.5- 7.4)	11.5 (8.9- NE)
Trastuzumab deruxtecan	DESTINY-PanTumor02	NCT04482309	II	HER2 2/3+ solid tumors, ≥ 1 prior line	41	NA	All: 39.0% (24.2-55.5) HER2 3+: 56.3% (29.9 to 80.2) HER2 2+: 18.8% (unknown CI)	All: 7.0 (4.2-9.7) HER2 3+: 7.4 (3.0-11.9) HER2 2+: 7.8 (2.6-11.6)	NA
Disitamab vedotin	RC48-C005	NCT03507166	II	HER2 2/3+, ≥ 1 prior chemo	43	NA	51.2 (35.5- 66.7)	6.9 (5.6- 8.9)	13.9 (9.1- NE)
	RC48-C009	NCT03809013	II	HER2 2/3+, ≥ 1 prior chemo	64	NA	50.0 (37.6- 62.4)	5.6 (4.1- 7.2)	14.2 (9.7- 18.8)
Datopotamab deruxtecan	TROPION-PanTumor01	NCT03401385	I	≥ 1 prior line	40	NA	27.5 (14.6- 43.9)	6.9 (2.9- NE)	NA
FGFR Inhibitors									
Erdafitinib	BLC2001	NCT02365597	II	FGFR2/3 altered, ≥ 1 prior line	99	NA	40.0 (30.0-49.0)	5.5 (4.2- 6.0)	13.8 (9.8- NE)
	THOR	NCT03390504	III	FGFR2/3 altered, 1-2 prior lines (including CPI)	266 (cohort 1)	Chemotherapy^*^	45.6 (37.1-54.3)	5.6 (4.2- 6.0)	12.1 (10.6-15.2)
				FGFR2/3 altered, 1 prior line, CPI naïve	351 (cohort 2)	Pembrolizumab	40.0 (32.7-47.6)	4.4 (3.7- 5.6)	10.9 (9.2-12.6)
Infigratinib	NA	NCT02150967	II	FGFR2 altered, 1-2 prior lines	108	NA	23.1 (15.6- 32.2)	7.3 (5.6- 7.6)	12.2 (10.7- 14.9)
Pemigratinib	FIGHT-201	NCT02872714	II	FGFR/FGF altered, ≥ 1 prior line or platinum eligible	103 (A-ID cohort)	NA	23.3 (15.5-32.7)	4.3 (3.9- 6.1)	8.9 (7.5- 15.2)
Rogaratinib	FORT-1	NCT03410693	II/III	FGFR 1-3 mRNA overexpression or FGFR3 mutation/translocation, prior platinum	175	Chemotherapy^*^	20.7 (12.7-30.7)	2.7 (1.9- 4.2)	8.3 (6.5- NE)

^*^chemotherapy: docetaxel, paclitaxel, vinflunine.

CPI, checkpoint inhibitor; FGFR, fibroblast growth factor receptor; HER2, human epidermal growth factor receptor 2.

Despite substantial therapeutic advances, most patients with mUC will ultimately experience disease progression either due to lack of initial response (primary resistance) or, more commonly, through the development of resistance after initial sensitivity (secondary resistance) (12, 13). These patterns of limited or transient efficacy are driven by intrinsic and adaptive tumor cell resistance mechanisms. Tumor cells can downregulate or modify target antigen expression, thereby reducing antigen-binding affinity. Following ADC internalization, enhanced lysosomal sequestration and overexpression of efflux pumps such as multidrug resistance 1-(MDR-1) and breast cancer resistance protein (BCRP) prevents ADC trafficking for payload delivery. Lastly, tumor cells can alter their own tumor microenvironment (TME), which can further attenuate therapeutic efficacy (**Figure 1**). Collectively, these resistance mechanisms highlight the need for combinatorial strategies and biomarker-driven approaches to overcome therapeutic escape and sustain durable responses.

Fibroblast growth factor receptor (FGFR) alterations are present in approximately 20% of mUC and are one of the few molecular targets with an FDA-approved therapeutic for mUC (14, 15). Erdafitinib, an FGFR 1–4 tyrosine kinase inhibitor (TKI), is approved for the treatment of mUC in patients harboring susceptive FGFR3 genetic alterations, underscoring the importance of routine somatic next generation sequencing in all patients with mUC. Several other FGFR targeting agents are under investigation. Ongoing trials continue to refine FGFR-targeting therapies to overcome resistance mechanisms, which highlights it role as a valuable therapeutic option for a subset of patients with mUC. (**Table 1**; **Figure 1**).

The advent of these novel therapeutics such as ADCs and FGFR-targeting TKI therapies has significantly expanded the treatment landscape for mUC. However, the targeted approach is not immune from the development of resistance and the key to improved long-term outcomes lies in identifying and circumventing these resistance mechanisms, both primary and secondary. This review explores the biological underpinnings of resistance to ADCs and FGFR inhibitors used to treat mUC and highlights emerging strategies aimed at circumventing these therapeutic challenges. This review provides the first systematic, side-by-side comparison of resistance mechanisms between ADCs and FGFR inhibitors in mUC, highlighting the overlapping and diverging pathways of therapeutic escape. By comparing these resistance patterns, this review can inform future development of combination regimens and drug design strategies to effectively overcome resistance.

## Antibody-drug conjugates of interest for treatment of urothelial carcinoma

### Enfortumab vedotin

EV is an ADC that is comprised of a human monoclonal antibody against nectin-4 conjugated to monomethyl auristatin E (MMAE), an inhibitor of microtubule formation, via a protease-cleavable linker (16, 17). Nectin-4 is a transmembrane protein that is highly expressed in multiple solid tumors, namely urothelial, gastric, and breast carcinomas, and is a marker of poor prognosis (18–20). When EV binds nectin-4, it becomes internalized and releases MMAE which disrupts microtubule formation and leads to cell-cycle arrest in tumor cells. Clinically, EV has shown promising efficacy in treatment of mUC. EV-201 was a single-arm, phase II clinical study of EV monotherapy that demonstrated a 52% overall response rate (ORR) in patients with mUC who had received previous CPI treatment without chemotherapy, highlighting the efficacy of EV in patients with limited treatment options (21, 22). The phase III trial EV-301 showed that EV monotherapy improved medial overall survival (mOS) (12.9 vs. 9.0 months; hazard ratio [HR] 0.70) and median progression-free survival (mPFS) (5.6 vs. 3.7 months; HR 0.62) compared to standard chemotherapy post-platinum and post-CPI over a median follow-up of 24 months. The ORR was 40.6% with EV compared to 17.9% with chemotherapy. Skin toxicity, hyperglycemia, and peripheral neuropathy are adverse events of particular interest when using EV.

EV leapt from the third line to the first line setting after EV-302 showed an improvement in OS in patients treated with EV plus pembrolizumab (EV-P) regardless of cisplatin eligibility (10). A new benchmark for median survival was set at 31.5 months vs 16.1 months with platinum doublet chemotherapy (hazard ratio 0.47; 95% CI 0.38 to 0.58, P<0.001). A total of 67.7% of patients responded to EV-P compared to 44.4% of patients who received chemotherapy; this includes a complete response (CR) rate of 29.1%. The improvement in survival was seen in both cisplatin-eligible and ineligible patients, a finding that has since made cisplatin eligibility criteria, largely in the first-line setting, a relic of the past (23). We know from an updated analysis presented at ASCO 2025 that of patients who achieved a CR, 74.3% of them maintained it 24 months later, indicating the durability of this response and opening the door to a potential cure in mUC (24).

### Trastuzumab deruxtecan

Human epidermal growth factor receptor 2 (HER2) is a transmembrane tyrosine kinase inhibitor involved in cell proliferation and differentiation that is overexpressed in many solid tumors including urothelial carcinoma (25, 26). Trastuzumab deruxtecan (T-DXd) is a HER2-directed ADC composed of an anti-HER2 antibody, topoisomerase I inhibitor payload, and a tetrapeptide-based cleavable linker (27, 28). In 2023, DESTINY-PanTumor02, a phase II trial, demonstrated that T-DXd had durable antitumor activity across many HER2 positive tumor types, including bladder carcinoma (n=41). The response rate was 56.3% in patients with UC with HER2 IHC 3+ and 35.0% in those with IHC 2+ (11). The tissue-agnostic approval of this agent in cancers with HER2 IHC 3+ has led to routine testing for this biomarker in the later line setting of mUC. Recent data from a phase Ib, non-randomized study showed that combining T-DXd with nivolumab, a PD-1 inhibitor, had synergistic anti-tumor activity in HER2-positive mUC (29). Pneumonitis remains an adverse event of interest with use of this drug.

### Disitamab vedotin

Disitamab vedotin (DV) is also a HER2-targetng ADC with a MMAE payload, like EV. Initially, DV monotherapy demonstrated potent antitumor activity in patients with HER2-positive locally advanced or metastatic UC who had progressed on at least one line of chemotherapy (30). In combined analysis of two-phase II clinical trials, DV monotherapy achieved an ORR of 50.5% of patients with HER2-positive, locally advanced or metastatic UC who had progressed on prior therapies (31–33). The median PFS was 5.9 months, and the median OS was 14.2 months. Beyond monotherapy, DV has been evaluated in combination with CPI to enhance antitumor activity (34–36). When combined with toripalimab in an HER2 unselected population within a phase Ib/2 trial, the ORR was 76% in treatment-naïve patients, 83.3% in those who were HER2 IHC 3+/2+, and even 33.3% in those who were completely negative for HER2 expression on IHC (32). An ongoing phase 3 study of DV plus pembrolizumab versus chemotherapy in patients with HER2-expressing mUC may further elucidate its role in the treatment of mUC (37).

### Sacituzumab govitecan

Sacituzumab govitecan (SG) is an ADC that is composed of an anti-trophoblast cell surface protein (Trop-2) IgG1 monoclonal antibody linked to an SN-38 payload. SN-38 is a metabolite of irinotecan, a topoisomerase I inhibitor (38). The phase II trial TROPHY-U-01 studied the efficacy of SG in patients with mUC who were heavily pretreated with both chemotherapy and immunotherapy. In the single-arm study, SG demonstrated an ORR of 28% with mPFS of 5.4 months and OS of 10.9 months. The study showed that SG had sustained clinical benefit independent of PD-L1 expression or prior response to checkpoint inhibition, leading to the accelerated FDA approval of SG for patients with mUC (39). More recently, TROPiCS-04 was a phase III randomized trial that compared SG to TPC (paclitaxel, docetaxel, or vinflunine) in the same patient population (40). Unfortunately, SG did not result in a statistically significant improvement in OS or PFS when compared to standard chemotherapy. However, the ORR was higher at 23% for SG compared to single-agent chemotherapy (14%). There was an enrichment in neutropenic deaths occurring within the SG-containing arm, most occurring within the first month of treatment, which may have at least in some part contributed to the negative results of this phase 1 study. Only 21% of patients received granulocyte colony stimulating factor (G-CSF) as primary prophylaxis was not mandated. This negative trial led to voluntary withdrawal of the accelerated approval status for SG by the manufacturer. Questions remain about the future of SG in mUC.

### Datopotamab deruxtecan

Datopotamab deruxtecan (Dato-DXd) is another ADC that targets Trop-2 with a topoisomerase I inhibitor payload. Trop-2 is overexpressed in several epithelial tumors, including UC, and is a marker of aggressive tumor behavior (41, 42). TROPION-PanTumor01 is an ongoing, phase 1, multicohort study investigating Dato-DXd in multiple tumor types and the early data from mUC was presented in 2025. A total of 40 patients were treated with the drug in the study, resulting in an ORR of 25%, disease control rate (DCR) of 77.5%, and a 6-month duration of response rate of 76.2%. This study was conducted in a heavily pre-treated population with 50% of patients having received at least 3 prior lines of therapy in the metastatic setting (43). Pneumonitis and stomatitis are the most concerning adverse events with this agent (44).

### Sacituzumab tirumotecan

Sacituzumab tirumotecan (Sac-TMT) is another Trop-2 targeting ADC linked to a topoisomerase I payload. A phase I trial of this agent was conducted in a patient population previously treated with prior platinum doublet chemotherapy and immunotherapy (45). In the patients who received sac-TMT in the second line (N = 11), the ORR was 45.5%; it was 26.3% in those which received it in the third line and beyond (N = 38). The primary adverse events that occurred were anemia (38.8%) and decreased neutrophil count (28.6%), however there were no treatment-related deaths to date.

## FGFR-3 inhibitors for treatment of urothelial carcinoma

Molecular studies of UC continue to identify new oncologic targets for targeted therapy. Among these targets, fibroblast growth factor receptor (FGFR) is an implicated oncogene that potentiates cell proliferation, differentiation, and angiogenesis via activation of the PI3K-AKT, PLC-gamma, STAT, and RAS-MAPK pathways (46). *FGFR* gene alterations, which have been identified in up to 20% of mUC and as high as 40% of cases arising from upper tract disease, are the biologic target for next-generation pan-FGFR inhibitors (15, 47). Erdafitinib is a potent FGFR 1–4 inhibitor that is now approved for the treatment of mUC in adults with susceptible FGFR3 alterations who have received at least 1 prior line of therapy. Unlike ADCs which are internalized within tumor cells, erdafitinib binds to surface receptor tyrosine kinase (RTK) to downregulate downstream aberrant signaling (48). This mechanism allows erdafitinib to exert its anti-tumor effect without relying on intracellular delivery, offering a complementary strategy to ADC-based therapies.

In a single-arm, phase 2 trial (BLC2001), 40% of patients with mUC with FGFR2/3alterations who progressed on platinum-based chemotherapy had an objective treatment response with erdafitinib monotherapy (49). The mPFS was 5.5 months (95% CI 4.2-6.0) and the mOS was 13.8 months (95% CI 9.8-not reached). THOR was the confirmatory phase 3 trial that compared erdafitinib to chemotherapy after tumor progression following anti-PD-1 or anti-PD-L1 therapy. The study found that erdafitinib resulted in significantly prolonged PFS of 5.6 months compared to 2.7 months in the chemotherapy cohort (HR 0.64; 95% CI, 0.47-0.88; p=0.005) (50). However, when compared to pembrolizumab monotherapy, erdafitinib had similar mean OS in FGFR-altered UC (51). Tolerability is a concern with FGFR inhibitors and patients on erdafitinib struggle with hyperphosphatemia, gastrointestinal symptoms, palmar-plantar erythrodysesthesia syndrome, and onycholysis. Erdafitinib is currently FDA approved for patients with mUC with susceptible FGFR3 genetic alterations after at least one line of prior systemic therapy.

Multiple other FGFR inhibitors have been investigated. A phase I trial of infigratinib, an oral FGFR 1–3 TKI in FGFR3-mutated mUC showed an ORR of 25.4%, mPFS of 3.75 months and mOS of 7.75 months in patients who progressed on at least one line of prior therapy (52). Pemigratinib is an oral GFR1–3 inhibitor that has been studied in a phase II, single-arm study in patients with FGFR3 mutations and other FGFR mutations. There was similar ORR in patients with susceptible FGFR3 mutations who received continuous dosing (23.9%) and intermittent dosing (24.6%). There was minimal activity in patients with other FGF/FGFR mutations other than FGFR3 (53). Rogaratinib is a pan-FGFR inhibitor (1-4) that was studied in a phase II FORT-1 trial that compared single-agent chemotherapy in patients with FGFR1/3 mRNA positive mUC with at least 1 prior line of therapy. The mOS was 8.3 versus 9.8 months (HR 1.11; 95% CI 0.71-1.72, P = 0.76) and the response rates between the arms were similar (20.7% versus 19.3%) (54). A major focus of ongoing FGFR targeting therapy is tolerability and more selective FGFR inhibitors are being investigated. One example is LOXO-435, an isoform-selective, small molecule inhibitor of FGFR3 designed to mitigate off-target effects of FGFR inhibition (55, 56).

## Mechanisms of resistance to ADCs and FGFR3 inhibitors

Because the mechanism of action of ADCs involves sequential molecular events to deliver its cytotoxic payload and disrupt DNA production, tumor cells can develop resistance mechanisms at multiple stages. These mechanisms include alteration of its surface antigens to diminish its binding efficacy, failure in drug internalization and trafficking, upregulation of drug-efflux pumps, resistance to cytotoxic payload, activation of bypass signaling pathways, and modulation of the tumor microenvironment (TME) (**Figure 1**). Further investigation into these mechanisms may provide a better understanding into why patients with mUC have insufficient responses to ADC therapy and FGFR inhibition.

### Antigen-related resistance

ADC and FGFR inhibitors target specific antigens on the surface of tumor cells. Therefore, a common proposed mechanism of resistance is tumor cell alteration of its surface antigen expression level. Loganzo et al. studied the mechanisms by which breast cancer cell lines develop resistance to ADCs through persistent drug exposure and found that downregulation of HER2 antigen or increased ABCC1 protein expression were the driving mechanisms (57). This preclinical study suggested that tumor heterogeneity in *HER2* expression may correlate to ADC efficacy and thereby to clinical outcomes. These results were corroborated in the KRISTINE and ZEPHIR clinical trials which found that tumors with increased heterogeneity in *HER2* expression prior to treatment with ADCs had worse clinical outcomes when compared to patients with tumors of low heterogeneity (58, 59).

To date, several studies have found that *NECTIN-4* amplification predicts EV response in mUC. Klümper et al. demonstrated that NECTIN-4 protein expression, independent of gene amplification, is downregulated during metastatic progression of mUC and that its expression correlates with EV response (60). This study suggests that NECTIN-4 amplification can be used as a genomic biomarker for patients with mUC undergoing EV therapy to predict clinical response. Similarly, alteration of target antigen is a common resistance mechanism against FGFR inhibitors. Gatekeeper mutations in FGFR3, such asV555M, following administration of FGFR inhibitors in non-small cell lung cancer can interfere with drug binding, leading to eventual erdafitinib resistance (61, 62).

### Impaired drug internalization and trafficking pathways

After binding to its surface antigen, ADCs are internalized into tumor cells via receptor-mediated endocytosis. ADC efficacy is reliant on target-mediated endocytosis to deliver its cytotoxic payload at sufficient concentrations to cause tumor cytotoxicity (63). There are several endocytosis pathways that mediate ADC uptake: clathrin-mediated endocytosis (CME), clathrin-independent endocytosis, and caveolae-mediated endocytosis (64, 65). CME is a receptor-mediated endocytosis process and is the most thoroughly studied in ADC transport. Briefly, the ADC-antigen complex gets internalized through clathrin-coated pits which invaginate and form vesicles with endosomes. The ADC is then trafficked through the endosomal-lysosomal pathway in which the cytotoxic payload is released. Sung et al. developed *in vitro* ADC-resistant breast cancer cells lines that internalize ADCs into caveolin-1 (CAV1)-coated vesicles which alters their trafficking to lysosomes (66). ADC colocalization into CAV1 vesicles reduced therapy response and was a negative biomarker for patient response. These results underscore the importance of understanding endocytosis pathways as alterations can impact therapeutic outcomes.

### Drug-efflux pumps and payload resistance

Another common mechanism of ADC resistance is elimination of the drug by the overexpression of ATP-binding cassette (ABC) transporters, which actively efflux the cytotoxic payloads out of the cancer cells. ABCB1 (P-glycoprotein) and ABCC1 (multidrug resistance-associated protein 1 [MDR1]) are the most well-characterized ABC transporters, which rely on ATP hydrolysis to translocate ADC payloads across cell members and thereby reduce its intracellular concentration. Corbett et al. showed that upregulation of ABCB1 and ABCC1 transporters was associated with resistance to ADC-containing pyrrolobenzodiazepine (PBD) and that inhibiting these transporters could restore drug sensitivity in previously resistant cancer cells (67). Similarly, T-DM1–resistant cells had an increased expression of ABC transporters despite preserved HER2 overexpression, and pharmacologic inhibition of these transporters reinstated T-DM1 responsiveness (68).

Upregulation of drug-efflux pumps correlates to enhanced toxic payload resistance. Studies have found that upregulation of drug efflux transporters such as P-glycoprotein is associated with resistance to MMAE, the payload used in EV. Chang et al. created EV-resistant bladder cancer cell lines *in vitro* through upregulation of P-glycoprotein and *TGF-β* genes which led to decreased sensitivity to MMAE (69). They found that resistance to EV was largely attributable to resistance to the payload MMAE rather than downregulation of surface antigen Nectin-4.

### Bypass signaling pathways

Activation of alternative signaling pathways is another mechanism of resistance to ADCs and FGFR inhibitors. One prominent mechanism involves the activation of PI3K/AKT/mTOR pathway which promotes tumor cell survival and proliferation. This pathway has been most prominently studied with trastuzumab which showed that PIK3CA mutations resulted in decreased sensitivity to the ADC, and that adding a PI3K inhibitor to trastuzumab had enhanced anti-tumor activity in HER2-positive metastatic breast cancer (70, 71). In mUC, upregulation of the PI3K/AKT/mTOR pathway correlates with increased EV resistance and enhanced tumor cell survival (72). Another critical bypass signaling pathway involves the TGF-β signaling pathway, which can induce the epithelial-mesenchymal transition (EMT) and enhanced tumor metastasis. In urothelial cancer, TGF-β signaling has been implicated in resistance to EV.

Similarly, resistance to FGFR3 inhibitors in urothelial carcinoma can occur through activation of downstream signaling pathways. Hosni et al. demonstrated that adipocyte precursor-derived neuregulin 1 (NRG1) promotes resistance to FGFR inhibition by activating the epidermal growth factor receptor 3 (ERBB3; also known as HER3) signaling pathway, which can bypass the inhibited FGFR3 pathway to sustain tumor cell proliferation (73). In another study, Weickhardt et al. showed that increased expression of phosphorylated ERBB3 is a key resistance mechanism in FGFR3-dependent bladder cancer and that dual targeting of FGFR3 and ERBB3 delayed the reactivation of pERBB3 and enhanced FGFR inhibitor efficacy (74). Better understanding of these signaling pathways is crucial in developing therapies that counteract resistance mechanisms and improve clinical outcomes.

### TME and immune evasion

The TME consists of network of stromal cells, immune cells, extracellular matrix, and soluble factors that interact with tumor cells. The TME plays an important role in tumor progression, metastasis, and immune escape which engenders resistance to ADCs. In addition to direct cytotoxicity, ADCs can induce immunogenic cell death (ICD), which is the release of damage-associated molecular proteins (DAMPs) such as calreticulin, ATP, and HMGB1 from dying tumor cells (75, 76). These DAMPs then activate dendritic cells (DCs) which in turn activate T cells to generate a robust antitumor response. Tumor cells can create an immunosuppressive TME phenotype through the recruitment of regulatory T cells (Tregs), MDSCs, and secretion of cytokines such as TGF-B. These changes decrease the effectiveness of immune effector cells, thereby inhibiting ICD and reducing the efficacy of ADCs. Recent studies have found that ADCs conjugated with payloads known to be strong modulators of the immune microenvironment such as pyrrolobenzodiazepine or tubulysin more effectively induce ICD, thereby synergizing with CPI (77, 78).

One significant mechanism involves upregulation of immune checkpoint molecules, such as programmed death-ligand 1 (PD-L1), which inhibits the cytotoxic activity of immune cells. This upregulation of PD-L1 can be a response to the inflammatory response induced by ADCs and creates an immunosuppressive TME, leading to an adaptive resistance mechanism. These changes decrease the effectiveness of immune effector cells, thereby reducing the efficacy of ADCs. As discussed above, combining ADCs or FGFR inhibitors with CPIs can potentially overcome this resistance mechanism.

Ouyang et al. explored the impact of FGFR3 alterations in bladder cancer TME and demonstrated that mutant FGFR3 indirectly induces an immunosuppressive TME by increasing serine synthesis. This activates the PI3K/Akt pathway and suppresses macrophage immunostimulatory functions, shifting them toward an immune-inert phenotype (79). Targeting PI3K in FGFR3 tumors reversed the macrophage phenotype and demonstrated synergistic antitumor activity when combined with erdafitinib. Overcoming these immunosuppressive modulations of TME through direct inhibitors is a potential strategy to enhance the effectiveness of ADC and FGFR3-inhibitors in mUC.

## Overcoming resistance to optimize ADCs and FGFR3 inhibitors

### Combination therapies

Overcoming tumor resistance to ADCs and FGFR3 inhibitors to increase clinical response rate requires a multifaceted approach. One promising strategy is the use of combination therapies. Combining ADCs with ICI both leverages the cytotoxic effects of ADCs while also activating the host’s immune system to target UC tumor cells. EV was initially approved as a single agent in the third line setting of mUC after prior platinum and prior CPI therapy. EV-302 study, a phase III randomized clinical trial, showed that when combined with pembrolizumab, EV had an overall response rate (ORR) of 67.7% compared to 45.2% in EV monotherapy arm in patients with untreated la/m UC (10, 80). These results highlight the synergistic and not just additive mechanism of action between EV and pembrolizumab and led to a change in the standard of care management of mUC (10, 81). However, despite these promising results, it remains uncertain whether the observed benefits represent true synergy or reflect additive effects from two separate agents. Additionally, overlapping toxicities such as rash, peripheral neuropathy, and immune-related adverse events require careful management and may limit broader applicability in frailer populations.

SC is another ADC which has been studied in combination with CPI. Results of the JAVELIN Bladder Medley trial were presented at ASCO 2025, a phase 2 study investigating maintenance avelumab plus SG versus avelumab alone. The study showed improved PFS but there was no significant difference in OS at the time of this interim analysis and there was toxicity incurrent in the investigational arm consistent with the known profile of SG (82). These findings underscore that not all ADC–ICI combinations yield clear survival advantages, and further data are needed to determine whether improved progression data translate to durable, clinical benefit. There is an enrichment of Trop-2 expression in variant histology and ongoing trials in rare bladder histology are of interest. The SMART trial is investigating SG with or without atezolizumab in locally advanced unresectable or metastatic rare genitourinary cancers including small cell, squamous cell, and adenocarcinoma of the bladder (NCT06161532) (83). The phase 1 DAD trial showed that combination EV and SG is possible. Of the 23 enrolled patients, the response rate was 70% with a manageable safety profile once prophylactic G-CSF was added (84). The DAD-IO trial builds on this concept and is a phase 2 trial of combination EV, SG and pembrolizumab in the first-line setting (85).

Similarly, overcoming resistance to FGFR inhibitors can also be achieved through combination therapies targeting complementary pathways. The BISCAY trial combined durvalumab, a PD-L1 inhibitor, with FGFR inhibitors in mUC but did not show enhanced efficacy over durvalumab monotherapy (86). In contrast, the NORSE study evaluated the combination of erdafitinib with cetrelimab, a PD-1 inhibitor, in FGFR-altered mUC, which had a ORR of 54.5% and a 12-month OS rate of 68%, compared to an ORR of 44.2% and a 12-month OS rate of 56% for erdafitinib monotherapy (87). This study could be interpreted as suggesting that combining FGFR inhibition with ICIs may enhance therapeutic efficacy by reprogramming the TME to support antitumor immunity. Although, it is not clear whether the efficacy is simply additive because of the two classes of agents. Moreover, long-term toxicity profiles of these combinations remain incompletely characterized, particularly given overlapping risks such as hyperphosphatemia, ocular events, and immune-related toxicities. Lastly, targeting bypass signaling pathways that contribute to resistance is another effective strategy. The phosphoinositide 3-kinase (PI3K) pathway has been identified as a key determinant of resistance to FGFR inhibitors (88). Inhibition of PI3K with agents such as BKM120 has been shown to act synergistically with FGFR inhibitors, enhancing their efficacy in urothelial carcinoma cell lines harboring FGFR mutations (88–90). However, these findings are largely preclinical, and translating synergistic activity into clinical benefit will require further evaluation of combinatorial dosing and scheduling in future trials.

### Targeting the TME

One strategy to overcome resistance is reprogramming the immune-suppressive features within the TME. For instance, MDSCs, tumor-associated macrophages (TAMs), and regulatory T cells (Tregs) facilitate an immunosuppressive TME which limits drug efficacy. Targeting these immune-suppressive cells with immune-modulating therapies such as CSF1R inhibitors can deplete TAMs. Resistance to FGFR inhibitors is mediated by the TME through promotion of TAMs which can secrete factors that activate the FGFR pathway, leading to tumor cell survival. Another approach involves remodeling the extracellular matrix (ECM) which can act as a physical barrier to drug delivery. Enzymatic degradation of the ECM by hyaluronidase can enhance the penetration of DCs within the tumor (91, 92). Normalization of tumor vasculature through anti-angiogenic agents such as vascular endothelial growth factor (VEGF) inhibitors or EGFR tyrosine kinase inhibitors (TKIs), such as osimertinib, may be useful.

Moreover, the TME can influence the expression of drug efflux pumps and enzymes that deactivate the cytotoxic payload. Resistance mechanisms that increase drug excretion by overexpressing drug efflux pumps can be overcome by implementing direct inhibitors of these pumps. Tariquidar, an ATPase inhibitor of P-glycoprotein drug efflux pump, has been used in combination with chemotherapy to increase drug exposure in resistance cancers, including renal cell carcinoma (93, 94). While no studies yet exist combining ADCs with drug efflux pump inhibitors, Cabaud et al. demonstrated that resistance to an anti-nectin 4 ADC could be reversed by tariquidar in preclinical breast cancer model (95).

### Novel drug design

One of the most direct strategies to circumvent resistance to ADCs and FGFR inhibitors is structural modification of these drugs to create next-generation compounds capable of overcoming established resistance mechanisms. There are three components of ADCs that can be altered to prevent resistance: antigen targeting, improved linker technology, and payload delivery. Novel bispecific ADCs that target two tumor cell antigens can reduce the likelihood of resistance due to antigen loss in tumor cells with low antigen expression. Dual targeting of HER2 by biparatopic ADCs has been shown to enhance toxin delivery to breast cancer cells with significant intratumor heterogeneity of HER2 expression (96). Preliminary results from a phase I study on a HER2 bispecific ADC, zanidatamad zovodotin, showed an ORR of 28% with disease control rate of 72% across multiple cancer types (97). Additionally, new bispecific ADCs that selectively bind two different antigens, such as Trop2 and Nectin4, are being studied in UC to enhance its selectivity and minimize off target effects (98). In addition, novel technologies like the bicycle drug conjugate (BDC) may be helpful to this end. These molecules are tiny with a short plasma half-life, holding the potential for increased tumor exposure and decreased normal tissue activity. Zelenectide pevedotin is a BDC highly selective for Nectin-4 and linked to MMAE. This agent is currently being studied within the DURAVELO-2 phase 2/3 study of the agent as monotherapy as well as in combination with pembrolizumab versus platinum doublet chemotherapy (99).

Secondly, linker structure can be modified to further optimize ADCs. Recent advancements have led to the development of cleavable linkers that are sensitive to specific TME conditions that ensure the payload is released within tumor cells. Incorporation of hydrophilic XTEN polypeptides in linker design have resulted in ADCs with extended half-lives (100). Lastly, payload delivery can be modified. Li et al. studied how the concentration of payload release affects ADC efficacy and found that the higher rates of payload delivery correlated to enhanced killing of neighboring tumor cells that did not express the target antigen, also known as the bystander effect (101). Creation of dual-drug ADCs, ADCs that deliver two distinct cytotoxic payloads, has shown synergistic antitumor activity in UC mouse models (102–104). Ongoing research aims to optimize the potential of dual-drug ADCs to overcome resistance where single-drug ADCs have failed in refractory tumor types.

Development of next generation of FGFR inhibitors is currently ongoing. Resistance to these agents often arise from secondary mutations in the FGFR kinase domain such as gatekeeper mutations like V555M in FGFR3 that reduce drug binding. Futibatinib, an irreversible FGFR1–4 inhibitor, forms covalent bonds with the kinase and maintains efficacy even in patients with solid tumors that have acquired resistance to prior FGFR inhibitors (105). Similar to ADCs, dual-targeted inhibitors are being engineered that can bypass resistance of activating signaling pathways such as EGFR, P13K/AKT, and MAPK pathways. Dual targeting of FGFR3 and ERBB3 showed to overcome resistance of FGFR3-fusion driven bladder cancer (74).

## Conclusion and future directions

The therapeutic landscape of mUC has rapidly progressed with the development of ADCs and FGFR inhibitors which offer durable clinical benefit to patients with mUC who have limited treatment options. However, both primary and secondary resistance to these therapeutics limit the depth and durability of response to ADCs and FGFR inhibitors. Intrinsic tumor resistance—namely, antigen downregulation, impaired ADC internalization and trafficking, drug efflux pumps, and bypass signaling pathways—alongside TME-mediated immune suppression all contribute to eventual therapeutic failure. In response, the development of next-generation ADCs and novel combination therapies must be aimed at overcoming these resistance mechanisms by enhancing tumor specificity and mitigating toxicity.

Now that EV and pembrolizumab have become first-line standard-of-care for mUC, the paradigm in this disease has shifted from the old guard of platinum doublet chemotherapy followed by immunotherapy. While outcomes have improved dramatically, most patients will not be cured of their disease even today. A better understanding of resistance mechanisms to these agents and how to overcome them will be essential. Fortunately, we have a wide armamentarium of potential options including biomarker selected and unselected ADC agents and FGFR inhibitors in the pipeline that are poised to change the future of this disease. A proactive approach utilizing combination therapies and novel agents to increase responses and survival is ideal, but attention also remains trained on salvage therapies to recapture response in patients who have experienced progression of their disease.
