# Supplementary material for: Overcoming resistance in advanced urothelial carcinoma: mechanisms of escape from antibody-drug conjugates and FGFR3 inhibition
Source: Front Oncol. 2025 Dec 2;15:1654771. doi: 10.3389/fonc.2025.1654771 (PMC12705392; doi:10.3389/fonc.2025.1654771)
Supplement: Supplementary file 1 [file Table1.docx]

| **Resistance Mechanisms** | **Antibody-Drug Conjugate (ADC)** | **FGFR Inhibitors** |
| --- | --- | --- |
| Antigen Loss/Reduced Target Expression | Downregulation or loss of expression of the target antigen (NECTIN4, TROP2) reduces ADC binding and efficacy | Direct target loss is less common but can occur via loss of FGFR3 expression or tumor selection for FGFR3-negative clones |
| Bypass Signaling Activation | Activation/upregulation of parallel survival pathways (RTKs, PI3K/AKT, MAPK) or mutations in payload targets can negate payload toxicity | On-target gatekeeper FGFR mutations reduce drug binding, and off-target bypass by activation of EGFR, MET, PI3K/AKT, RAS/MAPK, or other RTKs |
| TME Modulation and Immune Evasion | Immunosuppressive TME (stromal barriers, altered vasculature, or proteases) can reduce ADC penetration and blunt ADC-induced immune effects | TME immunosuppressive factors foster resistance and engender bypass signaling activation |
| Drug Efflux Pumps | Upregulation of ATP-binding cassette (ABC) transporters pump cytotoxic payloads out of tumor cells leading to reduced intracellular payload concentration | Efflux is a less dominant mechanism for small-molecule FGFR TKIs; ABC transporters can reduce intracellular TKI levels, decreasing drug exposure |
| Impaired Drug Internalization and Trafficking | Defective ADC internalization or lysosomal processing impairs payload release | Not applicable |

**Table S1**: A comparative overview of key resistance mechanisms to antibody–drug conjugates (ADCs) and FGFR inhibitors in advanced UC, highlighting both unique and overlapping pathways of therapeutic escape.
